# A two-step automatic identification of contrast phases for abdominal CT images based on residual networks

**DOI:** 10.1186/s13244-025-01995-7

**Published:** 2025-06-27

**Authors:** Qianhe Liu, Jiahui Jiang, Kewei Wu, Yan Zhang, Nan Sun, Jiawen Luo, Te Ba, Aiqing Lv, Chuane Liu, Yiyu Yin, Zhenghan Yang, Hui Xu

**Affiliations:** 1 Department of Radiology, Beijing Friendship Hospital, Capital Medical University, Beijing, China; 2 Department of Radiology, Mianyang Maternity & Child Healthcare Hospital (Mianyang Children’s Hospital), Mianyang, China; 3 Department of Radiology, Peking University Third Hospital, Beijing, China; 4 Department of Radiology, Peking University Cancer Hospital, Beijing, China; 5 Department of Radiology, The Second Affiliated Hospital of Dalian Medical University, Dalian, China; 6 Department of Radiology, The First Hospital of Fangshan District, Beijing, China; 7 Department of Radiology, Beijing Zhongguancun Hospital, Beijing, China; 8 Wuhan branch, United Imaging Intelligence Co., Ltd., Wuhan, China

**Keywords:** Abdomen, Computed tomography, Contrast media, Quality control, Deep learning

## Abstract

**Objectives:**

To develop a deep learning model based on Residual Networks (ResNet) for the automated and accurate identification of contrast phases in abdominal CT images.

**Methods:**

A dataset of 1175 abdominal contrast-enhanced CT scans was retrospectively collected for the model development, and another independent dataset of 215 scans from five hospitals was collected for external testing. Each contrast phase was independently annotated by two radiologists. A ResNet-based model was developed to automatically classify phases into the early arterial phase (EAP) or late arterial phase (LAP), portal venous phase (PVP), and delayed phase (DP). Strategy A identified EAP or LAP, PVP, and DP in one step. Strategy B used a two-step approach: first classifying images as arterial phase (AP), PVP, and DP, then further classifying AP images into EAP or LAP. Model performance and strategy comparison were evaluated.

**Results:**

In the internal test set, the overall accuracy of the two-step strategy was 98.3% (283/288; *p* < 0.001), significantly higher than that of the one-step strategy (91.7%, 264/288; *p* < 0.001). In the external test set, the two-step model achieved an overall accuracy of 99.1% (639/645), with sensitivities of 95.1% (EAP), 99.4% (LAP), 99.5% (PVP), and 99.5% (DP).

**Conclusion:**

The proposed two-step ResNet-based model provides highly accurate and robust identification of contrast phases in abdominal CT images, outperforming the conventional one-step strategy.

**Critical relevance statement:**

Automated and accurate identification of contrast phases in abdominal CT images provides a robust tool for improving image quality control and establishes a strong foundation for AI-driven applications, particularly those leveraging contrast-enhanced abdominal imaging data.

**Key Points:**

- Accurate identification of contrast phases is crucial in abdominal CT imaging.
- The two-step ResNet-based model achieved superior accuracy across internal and external datasets.
- Automated phase classification strengthens imaging quality control and supports precision AI applications.

**Graphical Abstract:**

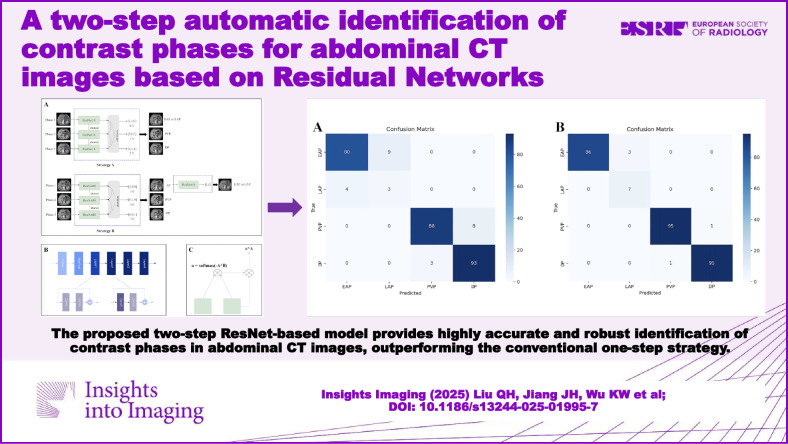

## Introduction

Liver diseases are diverse and represent the 11th leading cause of death globally, posing a significant health burden [1]. Abdominal CT, especially contrast-enhanced CT, is crucial for the detection and diagnosis of liver diseases [2–4]. For instance, arterial phase (AP), particularly the late arterial phase (LAP), plays a significant role in the detection and diagnosis of hepatocellular carcinomas (HCCs) because the enhancement of HCCs is usually marked in the LAP, and some HCCs show hyperenhancement only in this phase [5–7]. The portal venous phase (PVP) and delayed phase (DP) are valuable for detecting hypovascular lesions and observing late-phase enhancement or washout patterns [8]. Therefore, accurate identification of contrast phases is critical for abdominal CT quality control. Additionally, as each contrast phase reveals distinct characteristics of lesions, it is essential to select specific contrast phases based on the target task as image inputs when developing downstream artificial intelligence applications for medical imaging [9].

Although Digital Imaging and Communications in Medicine (DICOM) tags can provide some information about the timing of contrast phases, their accuracy is often limited by factors such as individual differences in patients’ circulation, discrepancies in naming conventions across different equipment manufacturers and human error. Technicians and radiologists can confirm whether the contrast phases are standard according to the enhancement features of the blood vessels and organs. However, these manual inspections are nevertheless time-consuming and labor-intensive. As a result, there is a need for automated methods to identify contrast phases, which can provide rapid and objective identification.

Deep learning has great potential and has achieved satisfactory performance in liver imaging, including CT and MRI, such as liver vessel segmentation [10, 11], detection and classification of focal liver lesions [12–14], staging of liver fibrosis [15, 16] etc. Therefore, the goal of this study was to develop and externally test a deep learning model based on Residual Networks (ResNet) for automatically identifying contrast phases in abdominal CT images.

## Materials and methods

This retrospective study was approved by the institutional Ethical Review Committee (Approval Number: 2022-P2-036-01), with a waiver of written informed consent.

### Dataset

**Fig. 1:**
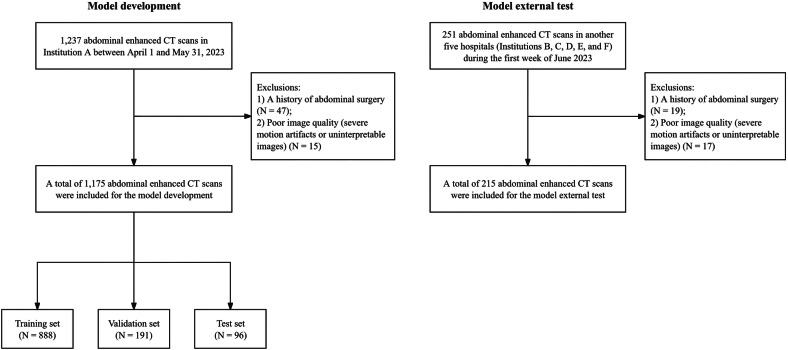
Flowchart of data acquisition, selection, and division

All abdominal enhanced CT scans were performed as part of routine clinical care for patients. For the model development, patients who underwent abdominal enhanced CT were retrospectively collected from Institution A between April 1 and May 31, 2023. For the model external test, a completely independent external test dataset was constructed, which retrospectively and consecutively acquired data of patients who underwent abdominal enhanced CT in another five hospitals (Institutions B, C, D, E, and F) during the first week of June 2023. Exclusion criteria included: (1) A history of abdominal surgery; (2) Poor image quality (severe motion artifacts or uninterpretable images). Each scan comprised three phases (AP, PVP, DP). The final cohorts included 1175 patients (3525 phase images, 1175 × 3) for development and 215 patients (645 phase images, 215 × 3) for testing (Fig. 1).

### CT acquisition protocol

All abdominal enhanced CT scans were performed using scanners from major manufacturers, including Philips, Canon, General Electric (GE), Siemens, and United Imaging Healthcare (UIH). Detailed CT scanning parameters are provided in Supplementary Table S1. Slice thickness was categorized as thin (0.625–1.5 mm) or thick (3.0–5.0 mm). Iohexol (Omnipaque 350, GE Healthcare; Ultravist 370 and 300, Bayer HealthCare) was injected into the median cubital vein at a rate of 3.0–3.3 mL/s using a power injector for abdominal enhanced CT axial scanning. The three different phases of each abdominal enhanced CT scan included the AP (25–40 s post-contrast injection), the PVP (60–75 s post-contrast injection), and the DP (140–155 s post-contrast injection).

### Annotations

All scans were anonymized. Two radiologists (with 7 and 9 years of experience in abdominal imaging, respectively) qualitatively labeled the actual timing of each enhanced phase according to the following criteria [17]: EAP = only strong enhancement in the hepatic artery, without enhancement in the portal vein and hepatic vein; LAP = moderate enhancement in the portal vein and liver parenchyma, without enhancement in the hepatic vein; PVP = strong enhancement in the portal vein, hepatic vein, and liver parenchyma; DP = the portal vein, the hepatic vein, and liver parenchyma are usually less enhanced in the DP than in the PVP.

When a disagreement appeared between the two radiologists, an expert (with 33 years of experience in abdominal imaging) participated in the discussion to reach a final consensus.

### Model development

**Fig. 2:**
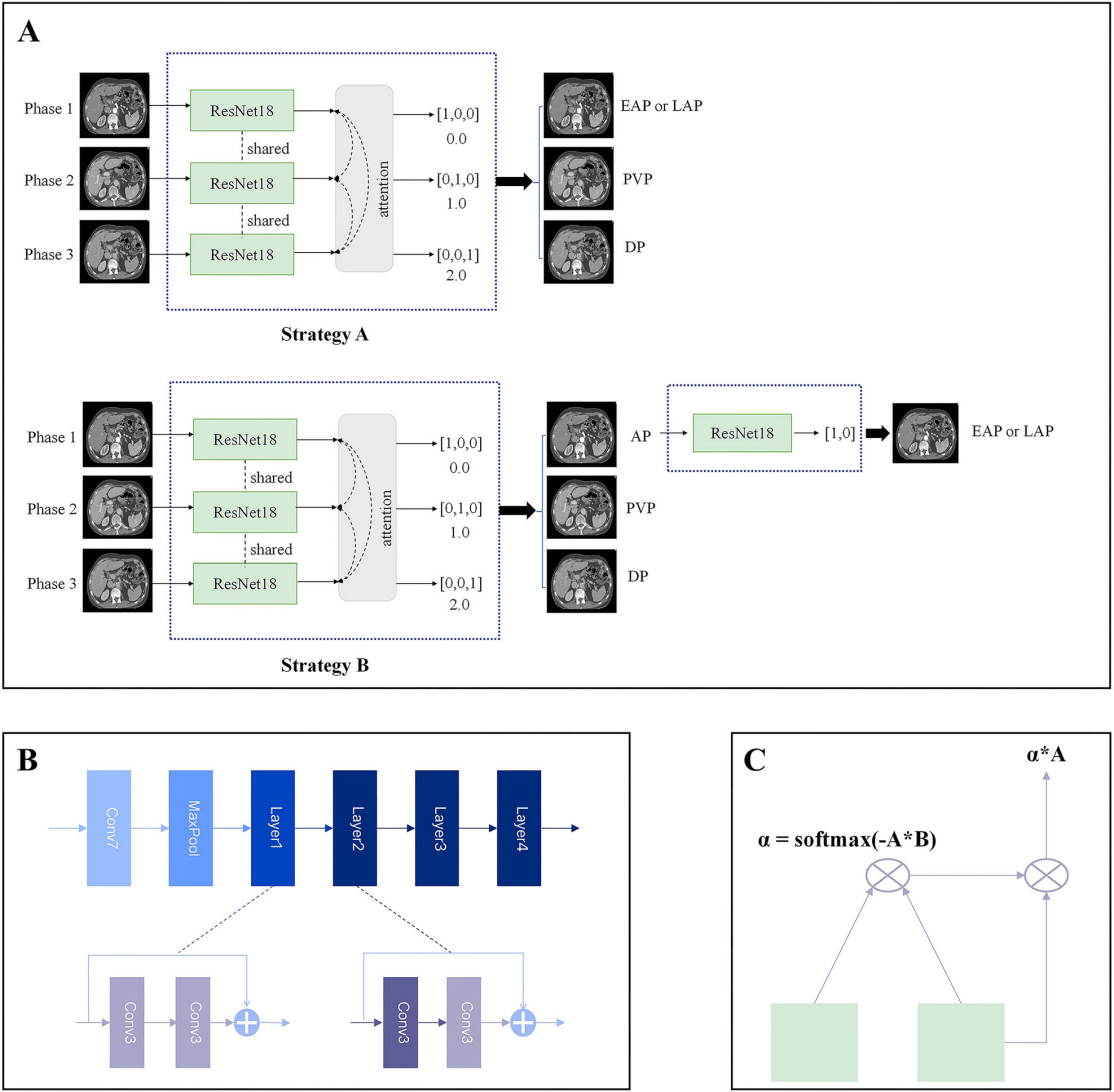
Overall structure and strategy of the model. **A** Schematic diagram of the model’s two contrast-phase identification strategies. **B** ResNet18 architecture of the model. The actual calculation method occurred after Layer 2 in Diagram B, where attention was calculated between the feature maps output from Layer 2, which then served as input to Layer 3. **C** Structure of the Attention Module

To develop a model that can identify EAP or LAP, PVP, and DP in one examination, we used Residual Network 18 (ResNet18) [18] as the shared feature extraction network for images. The last layer of the ResNet18 was a fully connected layer, and the activation function used was softmax. However, considering that the characteristics of the EAP and LAP were not significantly different, we proposed two strategies. Strategy A directly identified the EAP or LAP, PVP, and DP in one step. Strategy B had a cascading relationship and divided the contrast phase identification process into two steps. The first step was to identify the AP, PVP, and DP. Then, the second step further classified the images identified as the AP in the first step into either the EAP or LAP. The second step (sub-phase analysis between EAP and LAP) was activated only if the first-step output included AP. A conditional check ensured AP presence before initiating the second step. The architecture of the network used to identify enhanced phases is shown in Fig. 2.

The input data comprised three different contrast phases from each patient’s examination: AP, PVP, and DP, either thick-slice or thin-slice reconstructed images. During training, it also simulated cases where the number of phases was less than three. There was no need to input in a specific order; random input was acceptable. The data augmentation mainly involved random flipping. Multi-phase inputs were processed in parallel through ResNet18 with shared model parameters. This sharing mechanism referred to the parallel input of multiple phases in the model, where each parallel branch used the same set of model parameter. Meanwhile, to process multiple input data from different phases simultaneously, we incorporated an attention module. The attention module calculated attention weights, enhancing distinctions between high-dimensional features across different phases. By accentuating inter-phase differences, the model could focus on the unique characteristics of each phase. The attention weights were calculated as follows, using features from three phases:

$${{F^{\prime}} }_{AP}={F}_{AP}+{f}_{Attention}({F}_{AP},{F}_{PVP})+{f}_{Attention}({F}_{AP},{F}_{DP})$$

$${{F^{\prime}} }_{PVP}={F}_{PVP}+{f}_{Attention}({F}_{PVP},{F}_{AP})+{f}_{Attention}({F}_{PVP},{F}_{DP})$$

$${{F^{\prime}} }_{DP}={F}_{DP}+{f}_{Attention}({F}_{DP},{F}_{AP})+{f}_{Attention}({F}_{DP},{F}_{PVP})$$

Where F_*_ represented the feature maps associated with the respective enhanced phase, and F_*_′ represented the feature maps after attention calculation, ${{\rm{f}}}_{{\rm{Attention}}}$ could be calculated as:

$${f}_{{Attention}}(A,B)=A\times {\rm{softmax}}\left(-A\times B\right)$$

The data input was normalized to a range of 0–1 using a window level (WL) of 50 and a window width (WW) of 600, and then resampled to the size of [192, 192, 192].

The output for each phase was a four-dimensional vector corresponding to the probability values representing different phases, with the sum of probabilities equal to 1. The outputs of this model included identifications and relative orders of contrast phases. Among these, phase identifications were the model’s core outputs. The relative orders of phases could be adjusted with prior knowledge to calibrate misclassifications in the model’s output.

The model was built on the PyTorch framework, mainly using the torch library. We trained the model for 1000 epochs using the Adam optimizer with a learning rate of 1 × 10^−^^5^, and we typically selected the epoch model with the highest accuracy on the validation set. Every 100 epochs, the learning rate decreased by 10 times. The loss used here was focal loss [19].

### Statistical analysis

Continuous variables were expressed as mean ± standard deviation (SD) or as the median and interquartile range, depending on the normality of the data. Categorical variables were presented as frequencies and percentages. To evaluate the performance, sensitivity, specificity, positive predictive value (PPV), negative predictive value (NPV), and the area under the receiver operating characteristic curve (AUC) were calculated. The χ^2^ test was used to compare differences among subgroups, and multiple inter-group comparisons were adjusted using the Bonferroni correction. All analyses were performed using SPSS (version 25.0; IBM) and MedCalc (version 19.0.7; MedCalc Software bvba). A *p*-value < 0.05 defined statistical significance.

## Results

### Dataset characteristics

Each patient who underwent abdominal enhanced CT examination had three different contrast phases. For the model development, a total of 1175 patients from Institution A who underwent abdominal contrast-enhanced CT examination with 3525 enhanced phases were enrolled and randomly divided into training set (*N* = 888), validation set (*N* = 191), and test set (*N* = 96). Images were acquired from five different manufacturers (Philips, *N* = 548; Canon, *N* = 329; GE, *N* = 270; Siemens, *N* = 16; UIH, *N* = 12).

For the model external test, 215 patients who underwent abdominal contrast-enhanced CT with 645 enhanced phases from another five institutions (Institution B, *N* = 32; Institution C, *N* = 45; Institution D, *N* = 37; Institution E, *N* = 68; Institution F, *N* = 33) were collected. Images were acquired from four different manufacturers (Philips, *N* = 71; GE, *N* = 98; Siemens, *N* = 29; UIH, *N* = 17).

The above information and more detailed characteristics are presented in Table 1. No significant differences were observed in the distribution of age, sex, or slice thickness across the three datasets; however, the distribution of manufacturers varied among the groups.

**Table 1:** Dataset characteristics

Characteristics	Model development			External test
	Training set	Validation set	Test set	
Total (patients/phases)	888/2664	191/573	96/288	215/645
Age (years)	51.4 ± 15.8	52.6 ± 14.8	51.6 ± 15.6	61.9 ± 14.9
Sex				
Female	457	101	43	103
Male	431	90	53	112
Manufacturer				
Philips	468	54	26	71
Canon	213	73	43	0
GE	185	60	25	98
Siemens	12	2	2	29
UIH	10	2	0	17
Slice thickness				
Thin (0.625–1.5 mm)	638	142	74	70
Thick (3.0–5.0 mm)	250	49	22	145

*GE* General Electric, *UIH* United Imaging Healthcare

### Strategies comparison

**Fig. 3:**
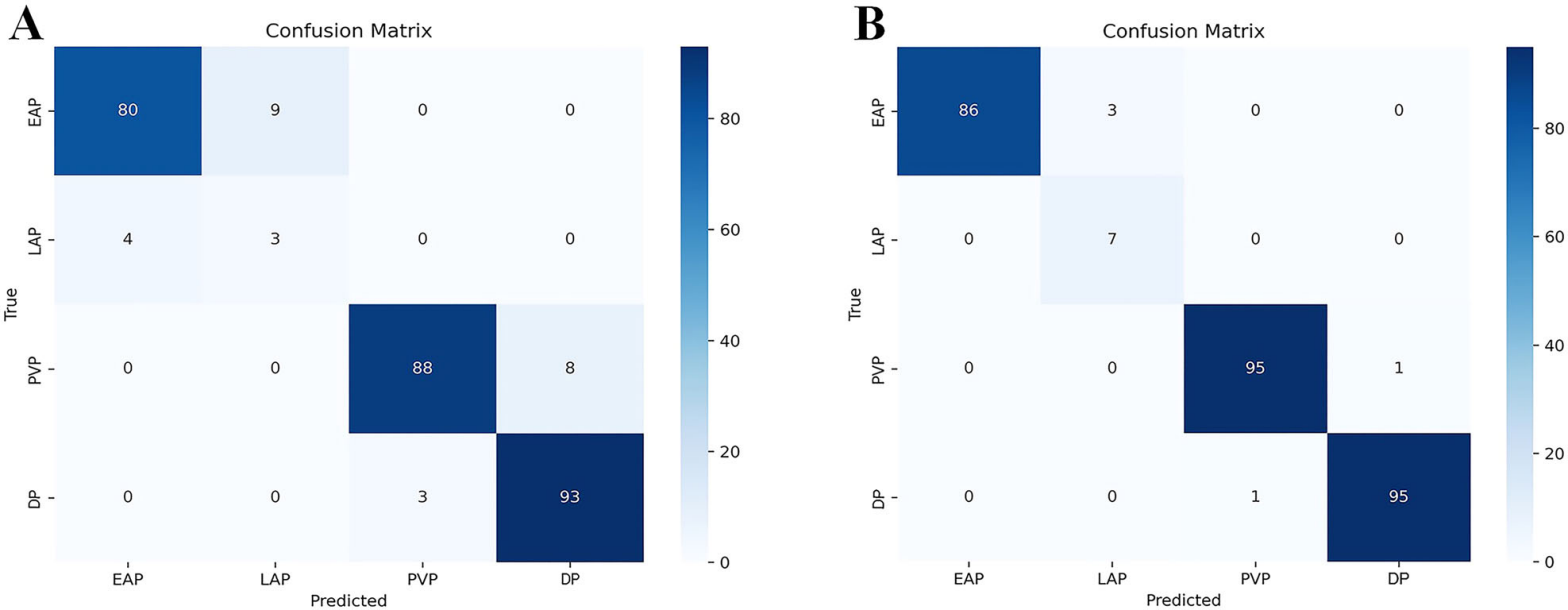
Confusion matrix diagram of the model in the internal test set. **A** One-step model performance. **B** Two-step model performance

In the internal test set, which consisted of 96 abdominal enhanced CT examinations and 288 enhanced phases, we compared the performance of the one-step classification strategy with that of the two-step classification strategy (Fig. 3). In phase-wise analysis, the overall accuracy for all enhanced phases of two-step classification strategy was 98.3% (283/288, 95% CI, 0.958–0.994), higher than one-step classification strategy (91.7%, 264/288, 95% CI, 0.877–0.945; *p* < 0.001). A two-step classification strategy outperformed one-step classification strategy in each enhanced phase (Table 2).

**Table 2:** The comparison of enhanced phase classification performance between one-step and two-step strategies in the internal test set

	Sensitivity (%)	Specificity (%)	Accuracy (%)	PPV (%)	NPV (%)	AUC	*p*-value
EAP							
One-step	89.9 (80/89) [81.2–95.0]	98.0 (195/199) [94.6–99.3]	95.5 (275/288) [92.4–97.3]	95.2 (80/84) [87.6–98.5]	95.6 (195/204) [91.55–97.8]	0.939 [0.905–0.964]	0.002
Two-step	96.6 (86/89) [89.8–99.1]	100.0 (199/199) [97.6–100.0]	99.0 (285/288) [97.0–99.7]	100.0 (86/86) [94.7–100.0]	98.5 (199/202) [95.4–99.6]	0.983 [0.961–0.995]	
LAP							
One-step	42.9 (3/7) [11.8–79.8]	96.8 (272/281) [93.8–98.4]	95.5 (275/288) [92.4–97.3]	25.0 (3/12) [6.7–57.2]	98.6 (272/276) [96.1–99.5]	0.698 [0.642–0.751]	0.003
Two-step	100.0 (7/7) [56.1–100.0]	98.9 (278/281) [96.7–99.7]	99.0 (285/288) [97.0–99.7]	70.0 (7/10) [35.3–91.9]	100.0 (278/278) [98.3–100.0]	0.995 [0.978–1.000]	
PVP							
One-step	91.7 (88/96) [83.8–96.1]	98.4 (189/192) [95.1–99.6]	96.2 (277/288) [92.4–97.3]	96.7 (88/91) [90.0–99.1]	95.9 (189/197) [91.9–98.1]	0.951 [0.919–0.973]	0.003
Two-step	99.0 (95/96) [93.5–99.9]	99.5 (191/192) [96.7–100.0]	99.3 (286/288) [97.5–99.8]	99.0 (95/96) [93.5–99.9]	99.5 (191/192) [96.7–100.0]	0.992 [0.974–0.999]	
DP							
One-step	96.9 (93/96) [90.5–99.2]	95.8 (184/192) [91.7–98.0]	96.2 (277/288) [93.3–97.6]	92.1 (93/101) [84.5–96.3]	98.4 (184/187) [95.0–99.6]	0.964 [0.935–0.982]	0.004
Two-step	99.0 (95/96) [93.5–99.9]	99.5 (191/192) [96.7–100.0]	99.3 (286/288) [93.3–97.6]	99.0 (95/96) [93.5–99.9]	99.5 (191/192) [96.7–100.0]	0.992 [0.974–0.999]	

Unless otherwise indicated, data are percentages and data in parentheses are numerators and denominators. Data in brackets are 95% CIs

*p*-value represents the difference in AUC between the two classification strategies in identifying each enhanced phase

*EAP* early arterial phase, *LAP* late arterial phase, *PVP* portal venous phase, *DP* delayed phase, *PPV* positive predictive value, *NPV* negative predictive value, *AUC* area under the curve

### Model external test

In the external test set, which consisted of 215 abdominal enhanced CT examinations and 645 enhanced phases, the comparison of the performance of the one-step and two-step models is provided in Supplementary Table S2. Given that the two-step model outperformed the one-step model, we opted for two-step classification strategy as the model architecture.

In the phase-based analysis, the overall accuracy for identifying enhanced phases was 99.1% (639/645). The two-step model performed best in classifying PVP and DP, followed by LAP. The sensitivity, specificity, accuracy, and AUC of the model for classifying EAP, LAP, PVP, and DP are shown in Table 3.

**Table 3:** The performance of two-step classification model in identifying each enhanced phase in the external test set

	Sensitivity (%)	Specificity (%)	Accuracy (%)	PPV (%)	NPV (%)	AUC
EAP	95.1 (58/61) [85.4–98.7]	99.8 (583/584) [98.9–100.0]	99.4 (641/645) [98.4–99.8]	98.3 (58/59) [89.7–99.9]	99.5 (583/586) [98.4–99.9]	0.975 [0.959–0.985]
LAP	99.4 (153/154) [95.9–100.0]	99.4 (488/491) [98.1–99.8]	99.4 (641/645) [98.4–99.8]	98.1 (153/156) [94.0–99.5]	99.8 (488/489) [98.7–100.0]	0.994 [0.984–0.998]
PVP	99.5 (214/215) [97.0–100.0]	99.8 (429/430) [98.5–100.0]	99.7 (643/645) [98.9–99.9]	99.5 (214/215) [97.0–100.0]	99.8 (429/430) [98.5–100.0]	0.997 [0.988–0.999]
DP	99.5 (214/215) [97.0–100.0]	99.8 (429/430) [98.5–100.0]	99.7 (643/645) [98.9–99.9]	99.5 (214/215) [97.0–100.0]	99.8 (429/430) [98.5–100.0]	0.997 [0.988–0.999]

Unless otherwise indicated, data are percentages and data in parentheses are numerators and denominators. Data in brackets are 95% CIs

*EAP* early arterial phase, *LAP* late arterial phase, *PVP* portal venous phase, *DP* delayed phase, *PPV* positive predictive value, *NPV* negative predictive value, *AUC* area under the curve

**Fig. 4:**
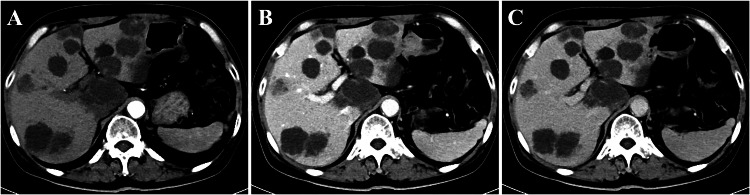
Abdominal contrast-enhanced CT in a 54-year-old female with liver metastases. **A** Early arterial phase (misclassified as late arterial phase by the two-step model). **B** Correctly identified portal venous phase. **C** Correctly identified delayed phase

In the examination-based analysis, there were 210 examinations in which all phases were correctly classified and 5 examinations with some misclassifications by the two-step model. Among the 5 examinations with misclassifications, the model misclassified EAP as LAP in 3 examinations (Fig. 4) and LAP as EAP in 1 examination. There was also 1 examination in which the model misclassified PVP as DP and DP as PVP. An accurate analysis of misidentified cases, based on measuring the intensity for each phase in both blood pools and parenchyma, is provided in Supplementary Table S3.

The performance of the model, stratified by sex, age, manufacturers, and slice thickness is presented in Table 4. No significant differences were observed among these subgroups.

**Table 4:** The phase-wise and examination-wise analysis of two-step model in the external test set

	Examination-wise		*p*-value*	Phase-wise		*p*-value^#^
	Classified correctly	Classified incorrectly		Classified correctly	Classified incorrectly	
Total	210	5		639	6	
Sex			> 0.99			> 0.99
Female	101	2		306	3	
Male	109	3		333	3	
Age			0.296			0.219
< 45	22	1		68	1	
45–59	81	3		250	4	
> 59	107	1		321	1	
Manufacturer			0.803			0.716
Philips	70	1		212	1	
GE	95	3		290	4	
Siemens	28	1		86	1	
UIH	17	0		51	0	
Slice thickness			> 0.99			0.670
Thin (0.625–1.5 mm)	68	1		206	1	
Thick (3.0–5.0 mm)	142	4		433	5	

*GE* General Electric, *UIH* United Imaging Healthcare

* *p*-value indicates the difference in examination-wise analysis

^#^ *p*-value indicates the difference in phase-wise analysis

## Discussion

In this study, we developed a deep learning model for the automatic identification of contrast phases in abdominal CT images using residual networks. This two-step model could classify contrast phases into EAP or LAP, PVP, and DP, achieving a high performance on both internal and external test sets and was not affected by the equipment from different manufacturers, demonstrating a robust generalizability.

Several previous studies [20–22] have focused on the automatic identification of contrast phases in abdominal CT scans. We summarized the methods, datasets, and other relevant details used in these studies in Supplementary Table S4. Dercle et al [20] developed a model to differentiate five contrast-enhancement phases based on a machine-learning algorithm and evaluated the model’s clinical applications, achieving accuracies of 90% and 84%, and 89.5% in identifying AP, PVP, and DP in the internal test set. In this study, they focused on the acquisition times of enhanced CT images and the density of the aorta and portal vein for contrast phase identification. However, due to various factors such as individual differences in patients’ circulation, human error, and inconsistency in entry, this information was neither sufficiently comprehensive nor reliable. Blankemeier et al [21] and Rocha et al [22] also developed a deep learning model to identify contrast phases in abdominal CT scans. The model developed by Blankemeier et al achieved accuracies of 89.5%, 81.9%, and 89.5% in identifying AP, PVP, and DP in the external test set, respectively. The model developed by Rocha et al achieved recall of 97.0%, 97.0%, and 94.0% for identifying AP, PVP, and DP in the internal test set, respectively. In their studies, contrast phase annotations were verified by radiologists to ensure the accuracy of contrast phase classification. However, these previous studies did not further differentiate between EAP and LAP. Therefore, we focused on further identifying the AP into EAP and LAP in this study, and the proposed two-step model achieved accuracies of 99.4%, 99.4%, 99.7%, and 99.7% in identifying EAP, LAP, PVP, and DP in the external test set, respectively, outperforming the results of the aforementioned studies.

To accurately differentiate between the EAP and LAP, we compared two strategies in the process of developing the model. One strategy was to directly identify contrast phases into EAP or LAP, PVP, and DP in one step. The other strategy involved a two-step process: initially identifying contrast phases as AP, PVP, and DP, and subsequently refining the identification of images identified as AP into either EAP or LAP. The results demonstrated that the two-step strategy achieved better performance. This may be because there were more pronounced differences in the enhancement characteristics of organs and vessels between AP, PVP, and DP, which allowed the model to effectively differentiate between these contrast phases. In contrast, the differences between the EAP and LAP were relatively minor, primarily involving the presence or absence of enhancement in the portal vein. In other words, the more classifications a model needed to perform simultaneously, the higher the performance requirements for the model became. Classifying four enhancement phases at once was more challenging than classifying two or three phases at a time. Therefore, decomposing the task into two simpler classifications effectively reduced complexity and improved model performance.

The detailed analysis revealed the following findings: in cases where the two-step model misidentified EAP and LAP (Cases 1–4), we observed that the portal vein exhibited slight enhancement during the AP compared to the pre-contrast phase, with an enhancement value of 10 + HU in most cases. Based on this observation, we hypothesized that the model’s excessive sensitivity to variations in the portal vein intensity led to these misidentifications. In the case where the two-step model misidentified PVP and DP (Case 5), we found no significant differences in enhancement between the PVP and DP phases in the portal vein and hepatic vein. The enhancement differences between PVP and DP were 34 HU for the portal vein and 9 HU for the hepatic vein. We speculated that the model’s error could be attributed to cirrhosis-induced intrahepatic structural distortion and hemodynamic alterations in this patient, which reduced the enhancement difference between the PVP and DP, leading to the model misinterpretation.

All abdominal CT images were preprocessed with uniform WW/WL settings (600/50). This setting was determined through comparative experiments evaluating multiple WW/WL combinations, including the routine abdominal setting (400/60), with selection based on validation performance. A potential limitation is the dual use of the validation set: for both selecting the best epoch for each model and identifying the optimal WW/WL combination. This approach may introduce selection bias and increase overfitting risk. Nevertheless, the model maintained strong performance on the multicenter external validation set, thereby reinforcing its generalizability. Additionally, the study has several other limitations. First, the model primarily focused on the enhancement features of organ structures within the same anatomical plane across different contrast phases. However, abdominal CT scans were subject to respiratory motion, and spatial registration at the image plane level might not guarantee accurate alignment of anatomical structures. This misalignment, in turn, impacted the model’s classification accuracy. Therefore, the model required further fine-tuning to ensure accurate registration between images from different enhanced phases before phase identification, which would help improve the model’s performance. Second, the model was only validated on retrospective datasets, both internally and externally, without prospective validation. While the model performed well on the retrospective datasets, the lack of prospective validation raised concerns about its real-world applicability and generalizability. Future research should focus on validating the model with prospective datasets to confirm its robustness and reliability. Third, the sample size of LAP used for the model development was relatively small, which might have contributed to the suboptimal performance of the model in identifying LAP. In future work, we plan to augment the LAP dataset to improve the model’s performance.

In conclusion, the proposed two-step model can accurately classify contrast phases of abdominal CT images into EAP or LAP, PVP, and DP in both internal and external test sets. This study offers an efficient and accurate approach for quality control in abdominal imaging. In addition, it also provides a robust foundation for the development of specialized AI tools, particularly those leveraging contrast-enhanced imaging series.

## Supplementary information

ELECTRONIC SUPPLEMENTARY MATERIAL

## Data Availability

All data generated or analyzed during this study are included in this article. Further enquiries can be directed to the corresponding author.

## Abbreviations

AP: Arterial phase
AUC: Area under the curve
DP: Delayed phase
EAP: Early arterial phase
GE: General Electric
HCC: Hepatocellular carcinoma
LAP: Late arterial phase
NPV: Negative predictive value
PPV: Positive predictive value
PVP: Portal venous phase
ResNet: Residual networks
UIH: United Imaging Healthcare
WL: Window level
WW: Window width
