# Supplementary material for: A two-step automatic identification of contrast phases for abdominal CT images based on residual networks
Source: Insights Imaging. 2025 Jun 27;16:139. doi: 10.1186/s13244-025-01995-7 (PMC12204963; doi:10.1186/s13244-025-01995-7)
Supplement: Supplementary file 1 — ELECTRONIC SUPPLEMENTARY MATERIAL [file 13244_2025_1995_MOESM1_ESM.pdf]

A two-step automatic identification of contrast phases for abdominal CT images based on Residual Networks

ELECTRONIC SUPPLEMENTARY MATERIAL

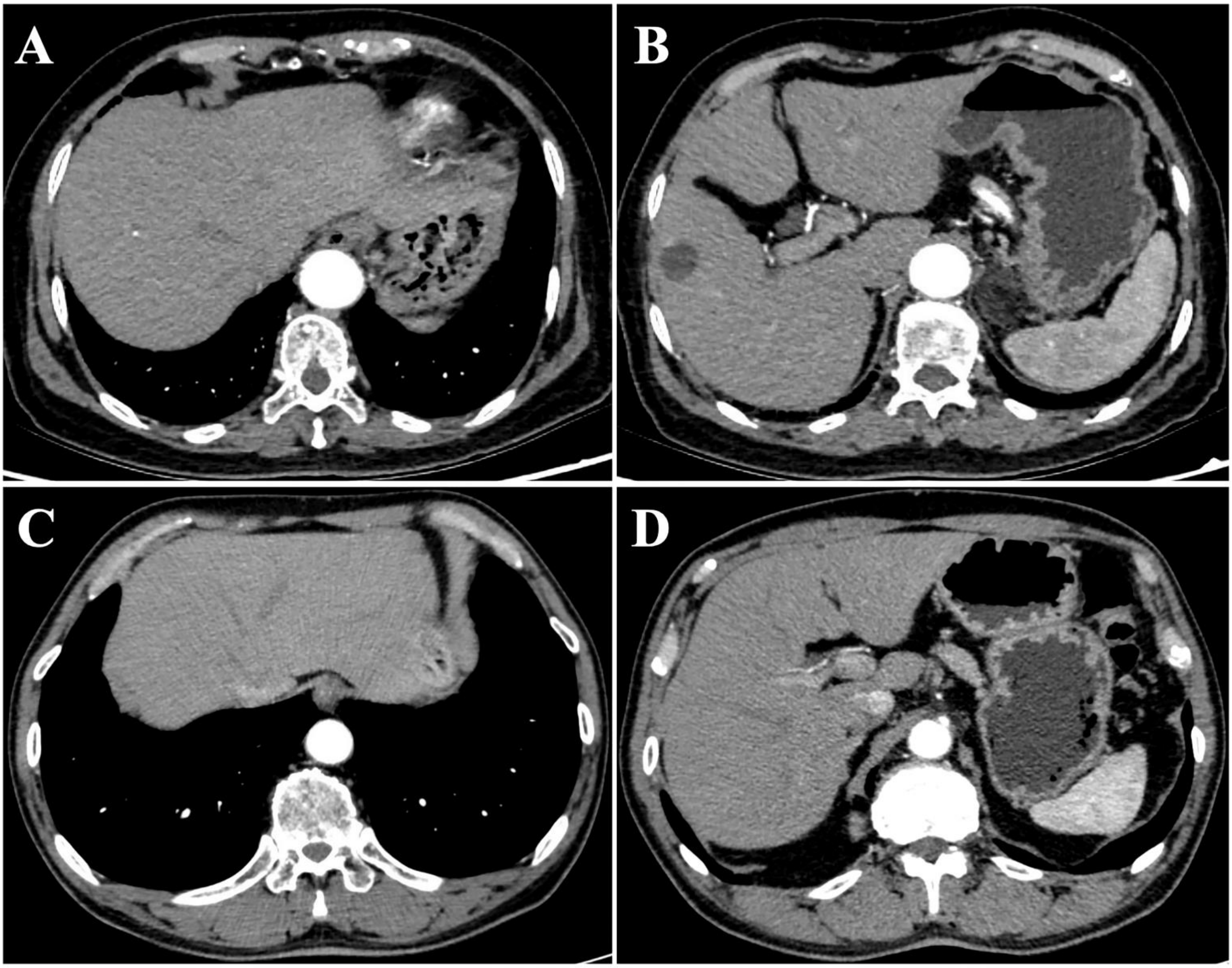

Fig. S1 Cases from the external test set in which the third radiologist participated. (A-B) A case interpreted as EAP based on the assessment of the third radiologist; (C-D) A case interpreted as LAP based on the assessment of the third radiologist

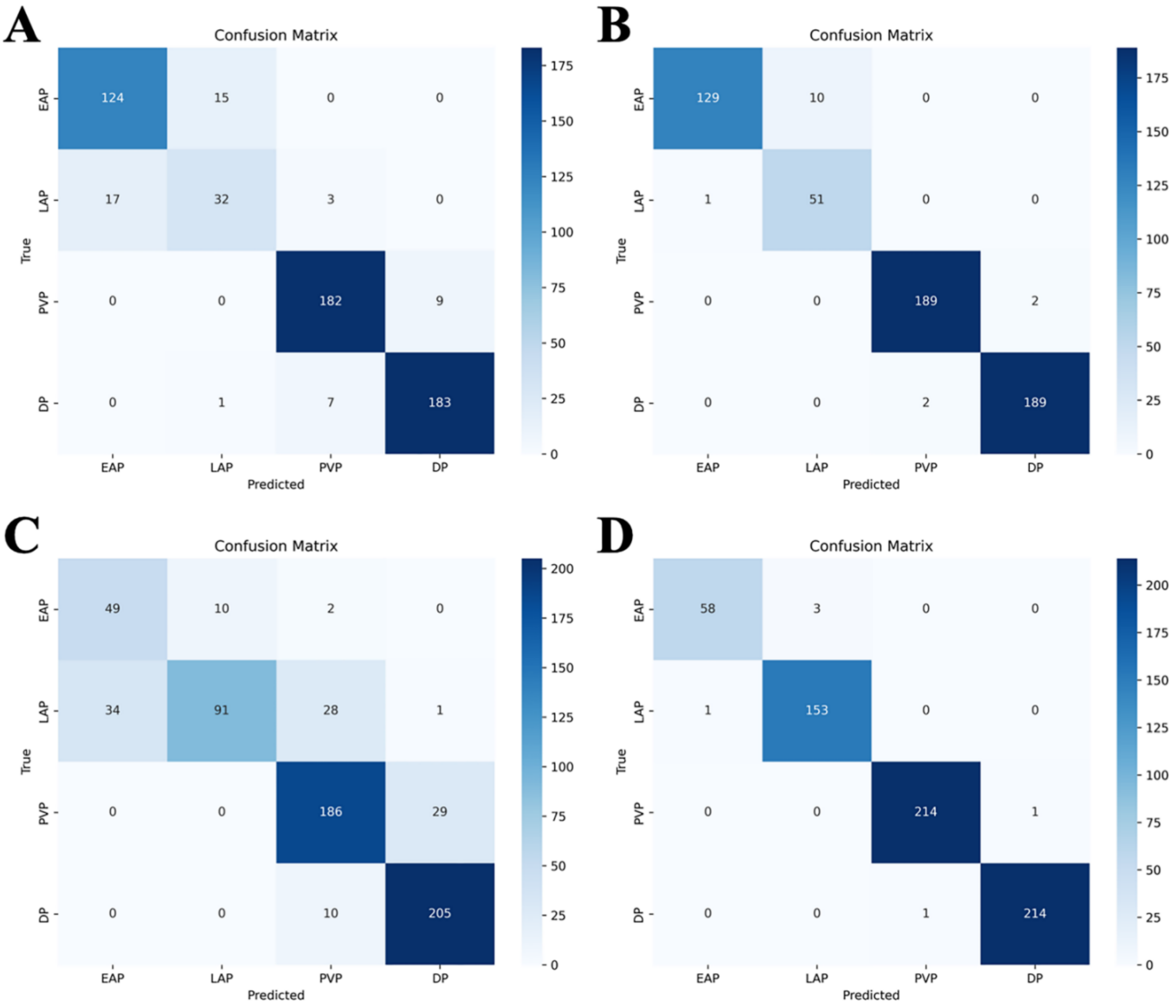

Fig. S2 Confusion matrix diagram of the model in the internal validation set and external test set. (A) Performance of one-step model in the internal validation set; (B) Performance of two-step model in the internal validation set; (C) Performance of one-step model in the external test set; (D) Performance of two-step model in the external test set

Table S1 The parameters for abdominal CT scanning

Manufacturer	Number	Machine	Detector (row)	Tube voltage (kVp)	Tube current (mA)	Rotation time (s)	Reconstruction kernel
Philips	619	Brilliance iCT (Philips Healthcare, Best, Netherlands)	128	120	400-750	0.6	Standard
		Brilliance 64 (Philips Health care, Best, Netherlands)	64	120	20-500	0.6	Standard
Canon	329	Aquilion ONE (Canon Medical Systems, Otawara, Japan)	320	120	50-900	0.5	Standard
GE	368	Revolution CT (GE Healthcare, Waukesha, WI, USA)	256	120	400-750	0.6	Standard
		Discovery CT 750HD (GE Healthcare, Waukesha, WI, USA)	64	120	375-600	0.5	Standard
Siemens	45	SOMATOM Definition Flash (Siemens Healthcare, Germany)	64	120	250	0.5	Standard
UIH	29	uCT960+ (United-Imaging Healthcare, Shanghai, China)	320	120	6-419	0.5	Standard

Notes: GE General Electric, UIH United Imaging Healthcare

Table S2 The comparison of enhanced phases classification performance between one-step and two-step strategies in the external test set

	Sensitivity (%)	Specificity (%)	Accuracy (%)	PPV (%)	NPV (%)	AUC	P value
EAP							
One-step	80.3 (49/61) [67.8-89.0]	94.2 (550/584) [91.9-95.9]	92.9 (599/645) [90.6-94.6]	59.0 (49/83) [47.7-69.5]	97.9 (550/562) [96.2-98.8]	0.873 [0.844-0.897]	< 0.001
Two-step	95.1 (58/61) [85.4-98.7]	99.8 (583/584) [98.9-100.0]	99.4 (641/645) [98.4-99.8]	98.3 (58/59) [89.7-99.9]	99.5 (583/586) [98.4-99.9]	0.975 [0.959-0.985]	
LAP							
One-step	59.1 (91/154) [50.9-66.9]	98.0 (481/491) [96.2-99.0]	88.7 (572/645) [85.9-90.8]	90.1 (91/101) [82.1-94.9]	88.4 (481/544) [85.4-90.9]	0.785 [0.752-0.816]	< 0.001
Two-step	99.4 (153/154) [95.9-100.0]	99.4 (488/491) [98.1-99.8]	99.4 (641/645) [98.4-99.8]	98.1 (153/156) [94.0-99.5]	99.8 (488/489) [98.7-100.0]	0.994 [0.984-0.998]	
PVP							
One-step	86.5 (186/215) [81.0-90.6]	90.7 (390/430) [87.5-93.2]	89.3 (576/645) [86.7-91.5]	82.3 (186/226) [76.6-86.9]	93.1 (390/419) [90.1-95.2]	0.886 [0.859-0.910]	< 0.001
Two-step	99.5 (214/215) [97.0-100.0]	99.8 (429/430) [98.5-100.0]	99.7 (643/645) [98.9-99.9]	99.5 (214/215) [97.0-100.0]	99.8 (429/430) [98.5-100.0]	0.997 [0.988-0.999]	
DP							
One-step	95.3 (205/215) [91.4-97.6]	93.0 (400/430) [90.1-95.2]	93.8 (605/645) [92.9-94.1]	87.2 (205/235) [82.1-91.1]	97.6 (400/410) [95.4-98.8]	0.942 [0.897-0.951)	< 0.001
Two-step	99.5 (214/215) [97.0-100.0]	99.8 (429/430) [98.5-100.0]	99.7 (643/645) [98.9-99.9]	99.5 (214/215) [97.0-100.0]	99.8 (429/430) [98.5-100.0]	0.997 [0.988-0.999]	

Notes: Unless otherwise indicated, data are percentages and data in parentheses are numerators and

denominators. Data in brackets are 95% CIs. EAP Early arterial phase, LAP Late arterial phase, PVP Portal venous phase, DP Delayed phase, PPV Positive predictive value, NPV Negative predictive value, AUC Area under the curve. P value represents the difference in AUC between the two classification strategies in identifying each enhanced phase

Table S3 The measurement results of enhanced CT values for the relevant blood vessels and organs in the erroneous cases of the two-step model in the external test set

	Hepatic artery (HU)	Portal vein (HU)	Hepatic vein (HU)	Inferior vena cava (HU)	Liver (HU)	Pancreas (HU)	Spleen (HU)	Renal parenchyma (HU)	Renal medulla (HU)
Case 1									
PRE	29	33	39	32	65	48	51	35	21
AP	282	46	36	38	68	78	75	80	44
PVP	130	140	134	89	95	81	94	138	90
DP	111	97	106	84	80	68	79	85	116
Case 2									
PRE	20	53	42	57	56	57	55	35	32
AP	271	66	43	75	64	97	81	183	53
PVP	143	171	156	146	81	95	118	182	86
DP	123	127	142	121	77	86	100	140	179
Case 3									
PRE	27	54	41	39	57	48	51	34	29
AP	422	69	38	82	67	132	87	177	73
PVP	182	222	151	102	117	141	148	244	85
DP	119	162	149	131	119	95	129	208	210
Case 4									
PRE	28	35	35	37	52	51	55	36	33
AP	264	138	37	68	54	117	135	153	75
PVP	138	168	161	101	94	92	111	170	86
DP	78	111	113	107	78	83	90	140	158
Case 5									
PRE	33	53	47	49	48	52	56	40	37
AP	257	136	49	103	58	123	118	189	85
PVP	112	155	118	125	83	86	116	172	105
DP	95	121	127	109	80	83	97	134	134

Notes: PRE Pre-contrast phase, AP Arterial phase, PVP Portal venous phase, DP Delayed phase

Table S4 The detailed comparison of previous related studies

Title	Methodology	Sample size	Performance	Publication year	Software	Dataset
Automated abdominal CT contrast phase detection using an interpretable and open-source artificial intelligence algorithm	They performed segmentation of five key anatomic structures—aorta, portal vein, inferior vena cava, renal parenchyma, and renal pelvis—using TotalSegmentator, a deep learning-based tool for multi-organ segmentation, and a rule-based approach to extract the renal pelvis. Radiomics features were extracted from the anatomical structures for use in a gradient-boosting classifier to identify four contrast phases: non-contrast, arterial, venous, and delayed.	200 patients/ 739 scans/ 1,545 series	In internal validation, the classifier achieved an accuracy of 92.3%, with an average F1 score of 90.7%. During external validation, the algorithm maintained an accuracy of 90.1%, with an average F1 score of 82.6%.	2024	The algorithm's public availability through the Comp2Comp Inference Pipeline, hosted on the GitHub repository "https://github.com/ StanfordMIMI/Comp2Comp".	Training with private data and validating on a public dataset "VinDr-Multiphase CT".
Contrast phase recognition in liver computer tomography using deep learning	They created an annotation platform and implemented a convolutional neural network (CNN) to automatically identify the CT scan phases (unenhanced, arterial, portal-venous and delayed) in the HCFMUSP database in the city of São Paulo, Brazil. They improved this algorithm with	396 patients/ 1,584 series/ 178.633 slices	It achieved an accuracy of 94.6%, 98% and 100% in the testing dataset for the slice, volume and exam evaluation, respectively.	2022	Private	Private

	hyperparameter tuning and evaluated it with cross validation methods.					
Using a single abdominal computed tomography image to differentiate five contrast-enhancement phases: A machine-learning algorithm for radiomics-based precision medicine	A random forest classifier was trained and tested to identify five contrast-enhancement phases. The input was the mean intensity of the abdominal aorta and the portal vein measured on a single abdominal CT scan image at a single time-point. The output to be predicted was: non-contrast [NCP], early-arterial [E-AP], optimal-arterial [O-AP], optimal-portal [O-PVP], and late-portal [L-PVP].	503 patients/ 3,397 time-points	The CECT-QC algorithm showed performances of 98 %, 90 %, and 84 % for predicting NCP, O-AP, and O-PVP, respectively.	2020	Private	Private
